# The complete chloroplast genome sequence of economical standard tea plant, *Camellia sinensis L.* cultivar Sangmok, in Korea

**DOI:** 10.1080/23802359.2020.1790311

**Published:** 2020-07-15

**Authors:** Dong-Jun Lee, Chang-Kug Kim, Tae-Ho Lee, So-Jin Lee, Doo-Gyung Moon, Yong-Hee Kwon, Jeong-Yong Cho

**Affiliations:** a Genomics Division, National Institute of Agricultural Sciences(NAS), Jeonju, South Korea; b Research Institute of Climate Change and Agriculture, Jeju-do, South Korea; c Division of Food Science, Chonnam National University, Gwangju, South Korea

**Keywords:** *Camellia sinensis* L, chloroplast genome, Sangmok, Korea

## Abstract

The complete chloroplast genome sequence of *Camellia sinensis L.* cultivar Sangmok was determined using high-throughput sequencing technology. We sequenced Sangmok chloroplast genome and performed comparative with 21 published other *Camellia* and species from different genus for phylogenetic analysis. Chloroplast genome was 153,044 bp in length, containing a pair of 24,627 bp inverted repeat (IR) regions, which were separated by small and large single-copy regions (SSC and LSC) of 19,155 and 64,665 bp, respectively. The chloroplast genome contained 97 genes (63 protein-coding genes, 29 *tRNA* genes, and 5 *rRNA* genes). The overall GC content of the chloroplast genome was 37.2%. The phylogenetic analysis among species in number of the genus *Camellia* provided that *C. sinensis L.* cultivar Sangmok is closely related to KJ806277 *Camellia pubicosta*.

Today, tea is commercially cultivated on more than 3.80 million hectares of land on a continent-wide scale, and 5.56 million metric tons of teas worldwide were produced annually in 2014. The *Camellia sinensis L.* cultivar Sangmok, which belongs to economical standard green Tea in Korea, is an evergreen small tree or shrub. Genus *Camellia* is an economic plant (Yang et al. 2013; Huang et al. 2014) including importantly well-known camellias (e.g. *Camellia japonica*, *Camellia reticulate*, *Camellia sasanqua*, *and Camellia oleifera*) (Ming and Bartholomew 2007; Meng et al. 2018) and it provides excellent materials for becoming increasingly popular owing to special health benefits and in plant science studies (Yang et al. 2013). The chloroplast genome information has been applied in plant biology, phylogenetic inference, and species identification (Kane et al. 2012; Ruhfel et al. 2014). In this study, we assembled the complete chloroplast genomes in *C. sinensis L.* cultivar Sangmok using Pacbio Sequel II sequencing technology. We have deposited the complete chloroplast genome sequence into GenBank database (Accession Number: LC488797).

The *C. sinensis L.* cultivar Sangmok was collected from the Research Institute of Climate Change and Agriculture in Jeju island, Republic of Korea (33.28N 126.31E) and registration of PVP Right (No 4837) in Korea Seed & Variety Service (KSVS, http://seed.go.kr/sites/seed_eng/index.do). We extracted genomic DNA from *C. sinensis L.* cultivar Sangmok leaves using SMRTbell Express Template Prep Kit version 2.0 (Pacific Biosciences, Menlo Park, CA) according to the manufacturer’ instructions. Library construction and sequencing were carried out using Pacbio Sequel II System at DNALINK Inc. (Seoul, South Korea). The data were produced using 152 Giga base pairs (Gbp) clean reads representing with a total of 128 Gbp of 10 kbp paired-end reads. We performed assembling of chloroplast genome by Celera Assembler 8.3rc2 and obtained 1 contigs. Chloroplast genome was confirmed by primer working method (Nadalin et al. 2012). We performed annotations from the KJ806281.1 *Camellia* species sequence.

The complement chloroplast genome of *C. sinensis L.* cultivar Sangmok was 153,044 bp length of circular form including four typical regions of chloroplast such as large single copy (LSC) region of 64,665 bp and small single-copy (SSC) region of 19,155 bp, separated by a pair of inverted repeats of 24,627 bp (IRa and IRb). The chloroplast genome contained 71 protein-coding genes, 37 tRNA genes, and 13 rRNA genes. The overall GC content was 37.2% containing a pair of 24,627 bp IR regions, which were separated by SSC and LSC of 19,155 and 64,665 bp, respectively. The chloroplast phylogenetic analysis was performed using previously published chloroplast genome sequences of 22 species belonging to Camellia family and different genus by neighbor-joining method in MEGA version 7.0 (Kumar et al. 2016) with 1000 bootstrap value (Figure 1). According to the phylogenetic tree, *C. sinensis L.* cultivar Sangmok was closely related to KJ806277 *Camellia pubicosta*, KF562708 *Camellia sinenis* var. Longjing, and KJ806278 *Camellia reticulata*.

**Figure 1.**
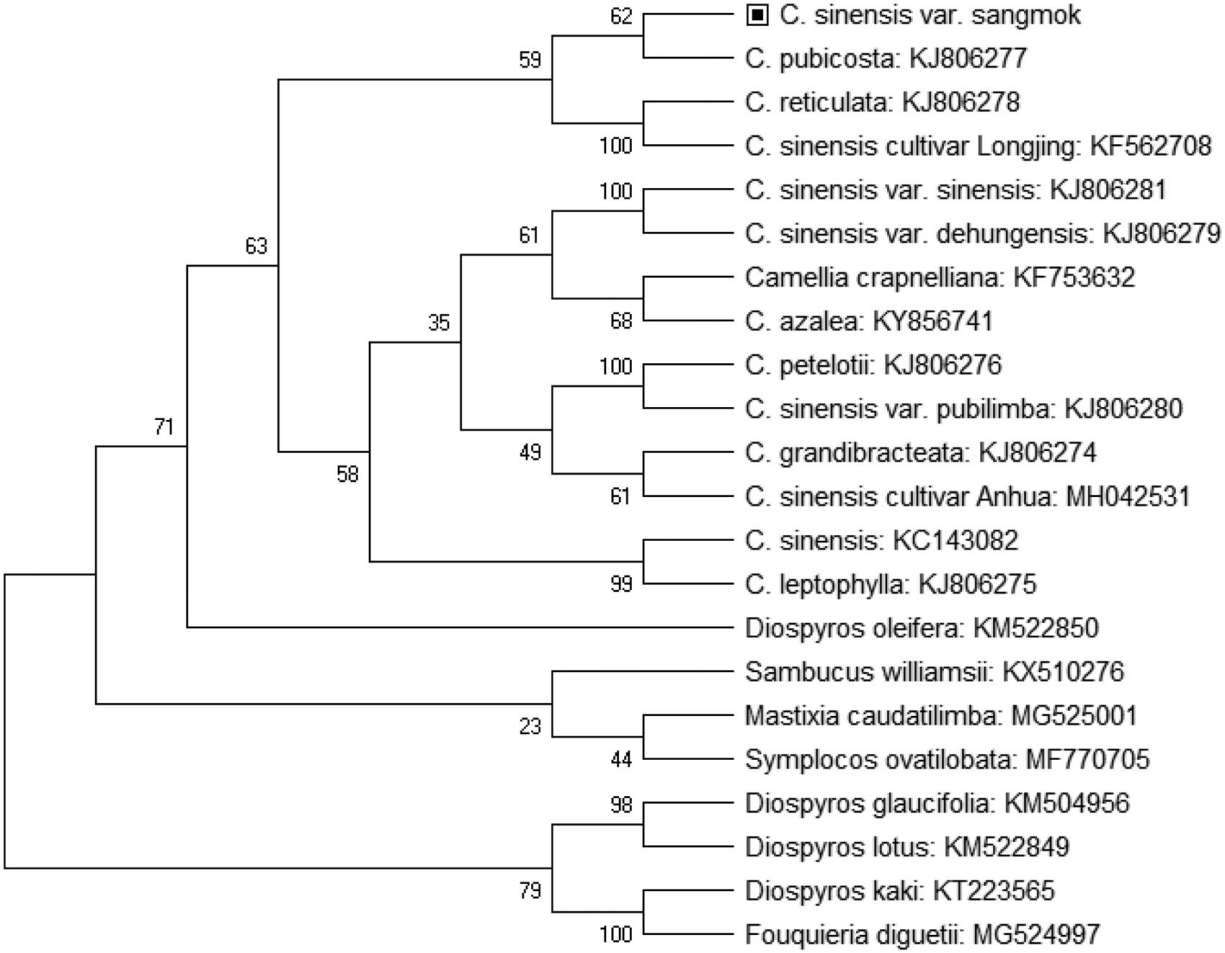
Neighbor-joining tree of *C. sinenesis* *L.* cultivar Sangmok and related species using chloroplast genome sequences. Numbers on the nodes are bootstrap values from 1000 replicates.

We expect that this sequence will help to clarify the hybrid status of *C. sinensis L.* cultivar Sangmok and provide additional genomic resources for *C. sinensis* studies.

## Data Availability

The data that support the findings of this study are openly available in GenBank of NCBI at https://www.ncbi.nlm.nih.gov, accession number LC488797.
